# Standards of Spelling, Etc., Adopted for the Buffalo Medical Journal

**Published:** 1913-10

**Authors:** 


### Standards of Spelling, Etc., Adopted for the Buffalo Medical Journal.

So far as practicable, the following rules are adhered to, although in original contributions, correspondence, etc., the same individual freedom is conceded as in the expression of opinions as to medical matters:

1. Excepting practical, technical, physical, clinical and the adjective chemical used as a noun by omission of the word substance, understood, Greek adjectives in -ic, are not lengthened by the Latin ending -al.

2. All Greek words beginning with R, were sounded with a preliminary H. In transcribing in Roman letters, this is written Rh, just as we write what and pronounce it hwat. This fact should not be forgotten in such words as Rhachitis, rhaphe, etc., nor should the doubling of the final r of syllables, by beginning the next syllable with rh, be forgotten, as in diarrhœa, nor even in catarrh, in which the rest of the syllable has been dropped.

3. As English is a very mixed language, which cannot be spelled phonetically except to a very limited degree, and then by arbitrary sound indicators, unless the alphabet is radically changed and increased to about thirty-six letters, we prefer to adhere to the established conventional method of using the ph instead of f, ch instead of k, œ and æ instead of e, etc., instead of the so-called simplified spelling, of classic words.

4. The established spelling instead of the simplified is also preferred in English words, as in writing the præterit and participal with -ed instead of -t.

5. The same word, unless a temporary compound or a chemic term in which a variety of suffixes are necessary to indicate special modifications, should not contain parts derived from different languages. Thus, amygdalitis should be written for tonsilitis, dodecalitis for duodenitis, proctitis for rectitis, splanchnoptosis for visceroptosis, superacid for hyperacid, etc.

6. Use regular forms instead of irregular so far as possible. Proved is not only preferable to proven for this reason, but because the verb was originally of the weak (regular) conjugation. The choice between English and foreign endings for the plural, etc., should depend upon how perfectly the word has been assimilated, and is, therefore, largely a matter of choice. If the foreign ending is chosen, be sure that the right form is used. Do not say spermatozoæ and remember that comitia and scyballa are already plural.

7. In regard to mood points in syntax, analyse the expression, remembering that English grammar, while very elastic, is almost absolutely logical. Do not say that far, but so far. Remember the distinction between no and not, and say whether or not. The Civil War decided the propriety of the expression, "The United States is," and any noun referring to a group may be considered singular or plural at will, providing that some consistency is observed and that we do not say that the laity or the profession is, in one sentence, and that the laity or the profession are in the next. But this latitude applies to verbs, not to demonstrative adjectives, so do not say "those kind." Moreover, the singularity or plurality of the verb must depend on the noun which is its subject and not on another noun connected to the former by of. We are on the fence as to whether none is strictly no one run together, or whether it is a collective and therefore may take a plural verb. The preceding sentence illustrates the view that there is no sense in requiring the repetition of an understood subject before each verb in a sentence merely because the verbs differ in mood, tense, etc. Don't let arbitrary authority blind you. "No body's else" is exactly as irrational as "Mr. Smith's Senior." There are many men who would rather split a fee than an infinitive. To avoid offense, we split infinitives as little as possible, but we can see no more objection to placing an adverb between to and the infinitive than between any other preposition and a gerund, or between an auxiliary and the principal verb, or to placing an adjective between a preposition and a noun.

8. While English is a very elastic and flexible language, don't stretch it too far in the endeavor to make one word do the work of several. When we read that Dr. A. operated a case and, if unsuccessful, posted it, it is a little too suggestive of such personal notes as that Mr. B. sundayed at East Nineveh.

---

We have had a few complaints about delay in publishing Mss. It should be understood that everything except news items is in the printers' hands on or before—sometimes two months before—the fifth day of the month preceding the date of issue. This is a physical necessity for all publications except those of a scale warranting the maintenance of an individual printing plant of considerable magnitude. Late in August we received Mss. absolutely promised in time for the July issue. The author made the promise in good faith, but important professional duties prevented him from fulfilling it. This is merely one illustration of the necessity of planning THE JOURNAL in advance. There are also other reasons, as seasonableness, desirability of grouping in one issue various articles dealing with related subjects, undesirability of printing in successive issues or those nearly related in time, papers on subjects recently treated or by authors recently represented, all of which reasons tend to delay publication for a few months.

Two years ago there was threatened a deficiency of original contributions, and we, therefore, planned, an issue in which the Abstract Department should be enlarged to fill a proportionate amount of space. The time for this special Abstract number has never arrived, but there are other reasons than a lack of original articles that favor the issue of such a number occasionally. We intend, therefore, to publish all Mss. on hand, as rapidly as possible; in fact, they are already in the printer's hands. This material will be exhausted by the February issue. Short articles can, however, be published earlier, and we shall not unduly delay the publication of longer communications for the sake of hurrying the Abstract number.

We would be greatly obliged for assistance in reviewing exchanges and in preparing abstracts. This request is not made for the sake of relieving the editor, since ordinary medical reading supplies an abundance of material for abstracts and the work of putting them into proper form for publication, instead of preserving notes for personal reference, is not very great. But work of this nature is of great value to the one who does it, and, by enlisting the services of a number of reviewers, a broadness of scope and of critical selection is assured, which renders the matter of greater value to readers generally. Many of the abstracts already published represent contributed work, which we acknowledge with thanks, and which would be indicated in the JOURNAL except for the modesty of the reviewers themselves. We do not wish to usurp credit due to others and shall be glad to publish contributed abstracts over the name or initial of the reviewer, if desired.

One of the best features of medical journalism, particularly in this country, is that the editors are mostly men engaged in actual practice. Some of the largest and most elaborate journals have been subject to criticism mainly because the view point of the editor was that of a man at a desk. The breadth of view resulting from daily contact with patients and the personal, continued experience with the exigencies of practice, is almost as necessary in the consideration of topics of ethics and professional polity as of strictly scientific and practical nature. But this great advantage cannot be secured without incurring the danger of lack of attention to minor details, and the more serious one of personal bias. The greater the number of individualities impressed on a journal, the more valuable is it. Even in matters of news the same holds good and the many favorable comments received as to this JOURNAL are due to the very loyal and extensive co-operation of its readers.

